# Towards Sustainable Distributed Sensor Networks: An Approach for Addressing Power Limitation Issues in WSNs

**DOI:** 10.3390/s23020975

**Published:** 2023-01-14

**Authors:** Alaa Alaerjan

**Affiliations:** Department of Computer Science, College of Computer and Information Sciences, Jouf University, Sakakah 72388, Saudi Arabia; asalaerjan@ju.edu.sa

**Keywords:** Contiki OS, distributed sensors, energy efficiency, IoT, sensors communication, wireless sensor networks

## Abstract

Distributed wireless sensor networks (WSNs) have been implemented in multiple applications. Those networks are intended to support the quality of operations and enhance applications’ productivity and safety. WSNs are constructed of a large amount of sensor nodes that are battery powered. Typically, wireless sensors are deployed in complex terrain which makes battery replacement extremely difficult. Therefore, it is critical to adopt an energy sustainability approach to enhance the lifetime of each sensor node since each node contributes to the lifetime of the entire WSN. In this work, we propose an approach to reduce power consumption in wireless sensors. The approach addresses power reduction in a sensor node at the sensing level, as well as the communication level. First, we propose configuring the microcontroller of the sensor to conserve energy based on the performed tasks. Then, we implement an interface to reduce consumed power by the radio module. Based on the approach, we carried out field experiments and we measure the improvement of power-consumption reduction. The results show that the approach contributes to saving up to 50% of the wasted energy at the sensor node and it improves communication reliability especially when the number of sensors in a network scales.

## 1. Introduction

The rapid development of information and communication technologies has created the potential for developing large-scale and distributed applications. The Internet of Things (IoT) has been built upon the concept of connecting physical (e.g., devices, sensors) and digital (e.g., applications, software) entities to improve automation, safety, and control [1]. The IoT notion aims at leveraging and enhancing exciting technologies to build interconnected applications, such as smart grids and smart cities. Monitoring different domains within IoT applications is a key aspect to ensuring their sustainability [2]. Wireless sensor networks (WSNs) are considered to be essential means for monitoring multiple aspects in IoT applications [3]. For example, WSNs are used in smart grids to monitor power domains to enhance quality of operations. They are also used in smart cities to improve quality aspects, such as safety and reliability. In fact, several IoT applications depend heavily on the utilization of WSNs since they play a fundamental role in enhancing the performance of those applications.

While the concept of employing wireless connectivity to span large geographical regions is not new, the introduction of low-power area networks (LoWPANs) has received significant attention in the last few years. This is due to the limitations in the lifetime of battery-powered devices, such as wireless sensors. Those devices depend greatly on radio technologies to communicate their data. Therefore, simple, robust, and narrowband modulation techniques are essential to enable the deployment of low-cost radio sensors with high receiver sensitivity. LoWPAN technologies, which include IPv6 low power area network (6LoWPAN) solutions offer a cost-effective solution to many IoT applications [4]. They are suitable since they provide wireless communication capabilities for supporting large-scale networks with numerous devices (e.g., smart metering) [5,6].

The improvement of hardware systems for the IoT is a critical study topic for 6LoWPAN researchers. Additionally, the development of low-power wireless communication systems is essential in this field of study. When building hardware and software for IoT devices (e.g., constrained wireless sensors), controlling power consumption is a major concern. This is because those devices are typically supplied by small capacity batteries to offer both sensing and actuation capabilities. In a sensor node, the radio is the source of a significant amount of power consumption [7].

Wireless sensors are designed to operate in a wide variety of different environments. Due to their intrinsic qualities and their flexible placements, sensors enhance the performance of monitoring of IoT applications. The sensor nodes that make up the network are anticipated to be small in size, reliable, low cost, and low power. Sensor nodes are often located in difficult-to-reach locations. Thus, their power is often provided by a battery or by harvesting energy from the surrounding environment (e.g., solar charging). Both these methods of power supply require management of consumption at their respective sensor nodes [4,8]. In the first method, it is necessary to prevent the battery from having to be changed. While in the second, it is necessary to keep the cost of the energy harvesting system as low as possible.

The medium access control (MAC) mechanism is the most important component of the data link layer. It is located above the physical layer, and it is responsible for controlling how common network resources are shared among connected nodes. Therefore, it is considered as a crucial control knob in any communication network. MAC must fully leverage the capabilities of the underlying physical layer without degrading the capabilities or the requirements of the application layer. When wireless IoT applications are being designed, typically simplicity and security are the most considered quality features. This is mainly the case where an application includes a large number of simple and autonomous devices that are deployed in the field to send data to the cloud [9]. In fact, distributed MAC solutions are often chosen because they cut down on the amount of traffic by not having to send control information to a single node with the objective of reducing network congestion.

The Contiki operating system (OS) is a lightweight OS that is designed to support wireless constrained devices in a network setting. Contiki OS may be used to administer 6LoWPAN platforms based on wireless internet communication technology. Typically, in wireless networks, it is required to relay data across multiple nodes in order to reach the intended destination. The radio duty cycle (RDC) protocol offered in Contiki OS enables end nodes and relay nodes to sleep and save energy between message transmission and relay. Consequently, sensor power consumption is improved, resulting in an improvement in the network lifetime.

The low-power-listening (LPL) mechanism is used to implement the ContikiMAC RDC protocol, which is included in the Contiki OS [10]. LPL makes use of the periodic WakeUp mechanism to constantly check the communication channel for any transmissions from nearby active nodes. In order to limit the amount of energy consumed by radio activities, ContikiMAC employs pairs of clear channel assessments (CCA) in every sleep and WakeUp sequence. Through the use of the received signal strength indicator (RSSI) thresholds, CCA is able to identify and distinguish between different types of interference [11,12]. The WakeUp is referred to be a single radio check that has a probability of detecting the presence of radio activity.

It is possible to divide WakeUps into three categories: Positive WakeUps, which result in the reception of the packet; False WakeUps, which result in noise or interferences; and Idle Listening, which is the case when the communication medium monitoring system detects no activity on the radio [13]. Due to the constraints of low-power radio, it is important to implement a CCA model on WSN hardware in order to check the radio activity. The radio listening mode employed by the receiver (Rx) consumes a significant amount of power. Therefore, it should be turned off once it is idle. On the other hand, it is recommended that nodes frequently listen to the radio channel in order to decrease communication latency in the network, even when data flow is sluggish [14]. The contradiction between these last statements shows the importance of power management in wireless sensors.

In this article, we propose a low-power solution for IoT WSNs using sensor nodes equipped with STM32 microcontrollers, S2LP radios, motion microelectromechanical (MEMS), and environmental sensor expansion boards. The proposed approach aims at reducing the power consumption of both the microcontroller unit and the S2LP radio module. The overall objective of the approach is to improve the lifetime of a WSN to ensure network sustainability. In the approach, we first configure the microcontroller unit to enter sleep mode in case of the absence of tasks to be performed. Then, we implement an interface allowing the use of ContikiMAC RDC mechanism to reduce the radio consumption. Given that, the CCA based on the computation of the RSSI is used to check the radio activity and, thereafter, decide whether to keep radio in the ON state until the reception of the packet or to put the radio in sleep mode. To prove the efficiency of the proposed approach, we carry out a comparative study with the case without RDC denoted by NullRDC.

Given the above discussion, we can summarize the contribution of the work into the following:

- Systematically defining the challenges that are encountered by several WSNs. This includes defining communication issues, as well as energy consumption factors at the wireless sensor level.
- Defining an approach for reducing energy consumption at the microcontroller unit in a sensor node. In this approach, not only the microcontroller energy is considered, but also the energy required by related peripherals.
- Defining an approach for reducing the required power by the radio module. In this approach, the notion of the useful active window for multiple communicating nodes is defined. Additionally, the time constraints and the synchronization aspects are illustrated to provide a comprehensive view of communication scenarios in WSNs.
- An implementation of the proposed approach to demonstrate its viability in reducing energy consumption in sensor nodes. This includes field experiments with multiple settings and configurations. The work also provides a set of comparison criteria to evaluate power reduction in the context of communication, complexity, and domain support.

The rest of the paper is organized as follows. Section 2 describes the importance of WSNs in IoT applications and the major challenges that are faced by wireless sensors. Section 3 highlights some of the related work. Section 4 describes the layout of the utilized network and the main characteristics of wireless sensors. Section 5 describes the main approach and illustrate the proposed power management techniques. Section 6 provides a use case where a simulation is developed to prove the viability of the proposed approach. Evaluation of results are described in Section 7. Finally, Section 8 concludes the paper and provides a discussion on the future work.

## 2. Background and Challenges

This section describes the role of WSNs in different IoT applications. It also highlights the limitations of wireless sensors nodes.

### 2.1. WSNs in IoT Applications

Different IoT applications, such as smart grids and smart cities, consists of a wide variety of WSNs [15]. WSNs are considered as key components in most IoT applications. Those networks are composed of a large number of sensor nodes, which are used to observe and communicate data about the surrounding environment. Sensor nodes have different functionalities ranging from safety-critical functions to providing trivial data sampling. However, all wireless sensor nodes are built with two basic functionalities, which are data sensing and data transmission. In fact, a WSN can be defined as a network with multiple nodes that cooperatively sense and communicate data with the objective to improve control in a given application [16].

Smart monitoring systems (SMS) are a key driver for enhancing quality of operations in IoT applications. Those systems depend heavily on utilizing wireless sensors, thus, WSNs are considered as the backbone in SMS [17]. Typically, SMS integrates a large number of intelligent devices that receive data from a variety of distributed sensors. In water production and water treatment plants sensor nodes are utilized to observe water contamination levels. The collected data from the sensors are sent to a control station through the network. Such data are considered critical; therefore, they are sent in real-time. The control station then makes decisions about whether the water is clean or contaminated. This complicated process relies greatly on the data that are collected and sent by sensor nodes, which shows the importance of WSNs in this IoT application (i.e., water monitoring system). According to Mumbi and Watanabe [18], water pollution cost is significantly high, and it may have other effects, such as health and environmental crises. It can be seen from the above example that WSNs play a crucial role in controlling key operations in IoT applications.

A smart grid (SG) is another important application of IoT [2]. SG is developed to replace the traditional power grid. It is designed to improve quality aspects, such as power efficiency and power reliability [19]. SG consists of multiple control stations. They make control decision based on the real-time data collected from different power domains. One of the main data streams that are used by control stations is sensor generated data. Multiple WSNs in SG are used to observe different aspects (e.g., power outage, power line overload, fault data) and relay information to control stations. For instance, in a power substation wireless sensor nodes are used to observe the status of a power transformers. If a fault has been observed (e.g., overheat, internal overload), the data have to be transmitted to a control device to perform a protective action. Wireless sensor nodes can also be used to observe the state of the power transmission lines; hence, rerouteing decisions can be made in case of a power overload. These automation examples depend mainly on the data generated by wireless sensors. In general, WSNs allow grid operators to monitor the state of the power grid in real-time to improve the automation process, which is one a core requirement in SG.

### 2.2. Wireless Sensors’ Challenges

Sensor nodes are the core components in WSNs. The combination of multiple sensor nodes with communication network forms a strong system that can be used to improve quality and efficiency in different IoT applications. The majority of sensors in a WSN are battery-operated devices. This means that the battery in a wireless sensor node is regarded as the most critical hardware component of a sensor device. Consequently, the batteries of wireless sensor nodes have a significant effect, not only on sensor devices, but also on the entire WSN. To illustrate, sensor nodes in WSN are distributed to cover different parts within the network. They relay data to each other and different data streams from different sensors form the overall status in a WSN. In case of energy depletion in a sensor, the node becomes non-operational. If multiple sensors in a given network suffer energy depletion, coverage holes may result, which affects the reliability and the correctness of the sensed data in the entire network.

**Figure 1:**
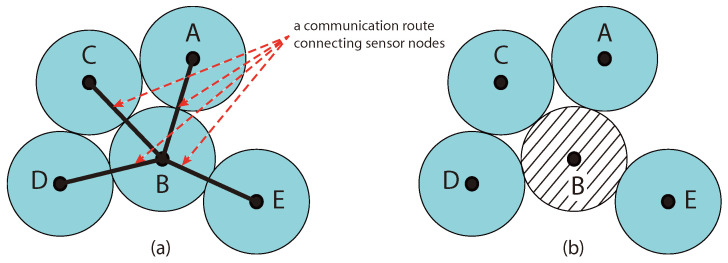
Sensor coverage hole example: (**a**) connected nodes, (**b**) communication route failure.

Energy depletion in a sensor node affects also the connectivity within a WSN. This is because WSNs are designed to allow sensor nodes to relay sensing data to each other to form communication routes [20]. Therefore, when a sensor node in a given route fails, the entire communication route becomes non-operational. Even if partial sensed data are extracted from a failed route, the reliability of the data cannot be ensured. A solution to overcome the failure in a communication route is utilizing alternative routes. However, such a solution may not be applicable based on the network topology, and if it is applicable, it may induce overhead in terms of energy and data latency. Figure 1a shows an example of five sensor nodes connected with a communication route. It illustrates that the sensor node in the middle *B* acts as a connection hub in the network. On the other hand, the graph (b) shows the problem when node *B* suffers energy depletion. This affects the entire network and causes two issues. The first is that the area covered by node *B* is no longer covered due to the failure caused by energy depletion. As a result, a coverage hole has resulted in the network. The second critical issue is the disconnection of the communication route, which causes the other sensor nodes to operate in isolation.

It is evident that power management in a sensor node is one of the crucial aspects that needs to be taken into consideration when designing WSNs. Energy consumption must be precisely managed in each sensor node within a network. This is required because the lifetime of a WSN depends greatly on the lifetime of the composed sensor nodes. In fact, the lifetime of a WSN is defined as the time duration that starts from running the network until the time that the first sensor node in the network depletes its battery [21]. Furthermore, managing power consumption of sensor nodes within a network is important since it affects other aspects, such as the security and reliability of the entire network [22]. Therefore, it is required to maximize the lifetime of a sensor node and achieve the optimal power utilization state.

There are a wide variety of WSNs that are used for different purposes. One example is the mobile WSNs, which consist of multiple sensor nodes that can be moved between physical locations. Such networks are designed to reorganize themselves based on the movement of the nodes to form a new topology. Terrestrial networks are another example of WSNs. Sensor nodes in terrestrial networks are organized either statically based on pre-defined manner (e.g., WSNs in an urban area) or based on an ad hoc manner, such as WSNs in remote locations (e.g., forests, deserts). Additionally, there is underwater WSNs, which are composed of multiple sensor nodes that are deployed under water. Those WSNs are utilized to observe underwater areas (e.g., deep ocean, contaminated water) that are not easy to reach otherwise. In general, a large number of WSNs are deployed in complex terrain where it is difficult to replace sensors’ batteries [23]. Hence, the only choice to keep the lifetime of a WSN for a rational amount of time is to manage the power on the composed wireless sensors.

## 3. Related Work

Due to the importance of WSNs, multiple researchers and organizations have been working towards addressing wireless sensors issues. A large amount of the work in the field of WSNs has been performed on enhancing the lifetime of battery-powered sensor nodes. Other works are proposed to address coverage holes to extend the lifetime WSNs. The following provides insights of the most relevant related works.

The authors Guo et al. [24] have proposed a method using protocol-based design. Their approach is based on dynamic channel assignment. More specifically, instead of broadcasting data, on a single channel, they propose partitioning the frequency band into multiple channels. Based on that, they propose assigning a channel to each node. The authors also introduce the notion of mobility with the objective of creating an adoptive protocol. They claim that their approach reduces the overhead of the network maintenance since it divides the frequency band into different channels. They also claim that their approach reduces power consumption since the radio on a sensor node can be turned off if the node is not transmitting data. However, such an approach may suffer scalability issues since it depends on channel partitioning.

Similar to the above, the work by Basagni et al. [25] uses a protocol-based design. Their work proposes an approach for addressing the limitation of WakeUp-based networks. It uses a collection tree protocol for data gathering in WSNs. The authors use the WakeUp receiver (WUR) to allow communication on transmit/receive to eliminate idle listening. Based on WUR, they enable the relay of WUR requests. The purpose of that is to extend the achievable range of WakeUp since the range of WUR is shorter than that of the main radio. Since the approach reduces the number of relay hops, the author clime that the lifetime of the entire network is improved. In fact, this approach depends on the size of the network, hence, it may not be suitable to large-scale WSNs.

The work by King and Roedig [26] proposes improving packet drop rate with the objective of reducing power consumption and improving communication. They propose increasing the threshold of CCA in order to reduce CCA collisions. They authors claim that by doing that the packet delivery rate in WSNs. Since packet drop rate is improved, power consumption is also improved. However, this approach depends on one CCA check, which makes it limited to specific protocols, such as Zigbee. In fact, any proposed solution to the issue of power consumption in sensor nodes should take into account that the majority of WSNs are heterogeneous. This means the solution should work around the principles of power consumption rather than addressing certain protocols or data models.

Some other works, such as [27,28], have targeted modifying the hardware in order to improve power consumption on sensor nodes. For instance, the work in [27] considered combining the emerging LoRa radio technology with energy harvesting WakeUp to create long- and short-range IoT nodes. Similarly, the work in [28] combines WakeUp and Bluetooth Low Energy (BLE) technology with energy harvesting. The proposed approach heavily depends on the utilization of hardware to allow the dual-radio mechanism to operate in a single sensor node. In fact, these approaches contribute to saving a considerable amount of power in a sensor node. However, they are considered to be complex since they require specific hardware and certain software management techniques.

The work by Tronci et al. [29] proposes a low-power WSN based on a multi-hop sensor data transmission technique. It employs low-power devices that communicate on the sub-GHz band. Data aggregation occurs by retransmitting packets to neighboring nodes. To ensure the consistency of data, the authors utilize time synchronization. The work also employs sleep mode technique to preserve power when the device is not in a communication mode. The authors have tested their work in a real-environment, and the results show improved power consumption, especially when devices are in sleep mode. However, the approach is vulnerable to coverage holes since it greatly depends on data retransmitting, especially when data routes are not carefully considered.

The authors in [30] propose a low-power wireless sensor node to monitor structural entities, such as bridges, buildings, and power towers. Particularly, they adopt Wireless Smart Sensors (WSSs) that is equipped with an accelerometer to read and transmit accelerometric measurements. Consequently, they develop WindNod to measure data that are induced by wind vibrations. In their approach, nodes communicate their data to a gateway, whose role is to store incoming data and resend it to the cloud for further processing. For communicating data, WSSs depend on BLE, which contributes to preserving power. However, the utilization of BLU limits the range of communication, which requires the network to increase the number of WSSs to extend data routes. Similarly, the work by Spencer et al. [31] proposes WSS platform to monitor the health of structural entities. The platform depends on power-optimized ZigBee communication to enhance power consumption, yet supports longer communication range. In their work, the authors adopt service-oriented architecture middleware to promotes software reusability and extendability. The objective of this work is slightly different since it focuses on multiple aspects, such as data management and data acquisition in sensor nodes and communication platform.

## 4. Network Layout and Wireless Sensors Characteristics

This section describes the network assumption and layout. It also describes the main energy consumption factors in a sensor node. Furthermore, it illustrates the platforms (i.e., software, hardware) that are used in this work.

### 4.1. Sensors Network Layout

**Figure 2:**
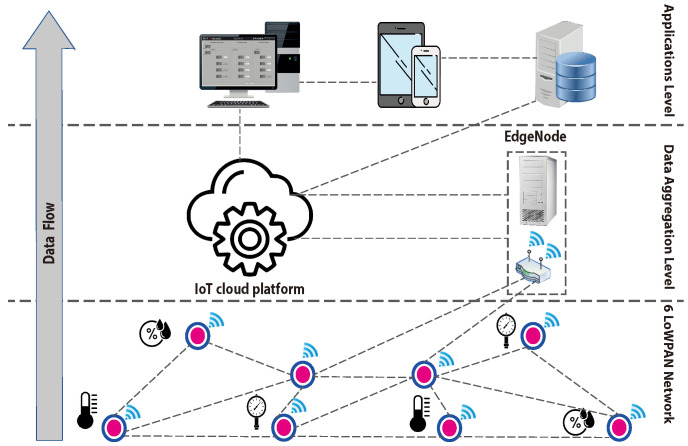
Architecture of considered IoT 6LoWPAN.

Figure 2 illustrates the layout of the wireless sensor network that is considered in this paper. It represents a 6LoWPAN network, which consists of several sensor nodes that are wirelessly connected to each other. The figure shows three virtual layers. The first is the field layer (i.e., 6LoWPAN network), which consists of the actual wireless sensors. In this layer different communication channels are usually established to provide wider coverage and fault-tolerance. In the second layer, data aggregation entities, such as edge nodes and cloud platforms (e.g., servers, cloud storage) are deployed. Those entities are placed in this layer to facilitate data collection and then transfer collected data to higher layers. The top layer is the application layer where data are processed and results are made available to end users. Multiple tasks such as data storage, presentation, and formatting are carried out at this layers. Control decisions are also made at this layer.

Typically, in this network setting, sensor nodes are connected to the Internet via a single-board router, since it has sufficient computing power to process multiple data streams [32]. The illustrated layout is common in several IoT applications, such as smart grids and smart cities. In a smart grid, there are several WSNs in the transmission domain [33]. Those networks are used to monitor equipment, such as power transformers and transmission lines. Similarly, a smart city consists of different types of low-power wireless sensors that are represented in a 6LoWPAN network. They are used for several purposes, such as monitoring water distribution networks and observing vehicles congestion. Since wireless sensors are limited to perform specific functionalities, their data are usually limited to few bytes. Hence, 6LoWPAN is suitable for handling the communication between those devices and intended destinations (e.g., cloud, control centers).

### 4.2. Sensor’s Energy Consumption Factors

The work in this paper aims at improving the sustainability of WSNs. Therefore, managing power on each sensor node within a network is a key factor since it contributes to the lifetime of the entire network. Given that, in this work we consider the three main sources of energy consumption in a wireless sensor node, and they are:

- The energy utilized by the microcontroller unit within a sensor node.
- The energy consumed by the radio module within a sensor node.
- The energy consumed by the RDC mechanism that is used to control the periodic WakeUps. Controlling this mechanism is essential since it affects both power consumption, as well as communication channels among neighboring nodes.

Based on these three factors, the following describe the proposed approach to reduce energy consumption on a sensor node.

### 4.3. Platforms

Over the past few decades there has been a significant effort to develop flexible operating systems to meet the challenging demands of WSNs. Typically, WSNs have their own characteristics, as well as challenges. For example, mobility of nodes is one of the characteristics that can be found in WSNs but not other types of networks. Additionally, WSNs might be constructed using heterogeneous sensor nodes. Furthermore, sensor nodes within WSNs have power consumption limitations [34]. Due to all these limitations and attributes, several operating systems have been developed specifically to be deployed in WSNs.

Contiki OS [35] is one of those operating systems. It is a light-weight operating system developed to manage low-power wireless devices, such as wireless sensor nodes. This operating system is considered open source and it is developed using the C programming language. Contiki OS is widely used in IoT applications since it allows building network-connected entities, such as wireless sensors and monitors. It also supports building web-based automation systems, which makes the operating systems suitable for applications that require remote monitoring and control. Given all the above described features, we use Contiki OS as the core platform in our work in this paper.

Constrained hardware devices require low-power radio module. S2LP is one of the well-known radio modules that is commonly used with wireless sensor nodes. S2LP is an ultra-low power radio frequency (RF) transceiver. The module is designed to provide high performance for RF devices, such as wireless sensors and wireless trackers. S2LP can operate in ISM (industrial, scientific, and medical) and SRD (short-range device) frequency bands at 433, 512, 868, and 920 MHz. It can be also configured and programmed to operate at other frequencies depending on the application domain. In this work, we use S2LP as the main communication interface (i.e., radio module) for all sensor nodes.

Due to the rapid development of wireless technologies, WSNs are constructed using a range of constrained sensor devices. Those devices are manufactured by different vendors. The majority of those sensors contain similar hardware components. They also follow similar operation principles. To illustrate, a wireless sensor node is composed of a microcontroller, a communication interface (e.g., radio, bluetooth module), and a battery. In this work, we use the STM32 as a microcontroller to describe the proposed power reduction approach. The STM32 microcontroller has been chosen since it is commonly used in several WSNs. It is also used in other IoT applications, such as low-power health applications (e.g., wearable healthcare monitors, patient medical equipment) and smart home applications (e.g., smart appliances).

Table 1 shows a comparison between commonly used sensor hardware components (i.e., microcontroller) in IoT and industrial applications. The table provides further details illustrating why STM32 microcontroller has been chosen in this work. STM32 is compared to other hardware such as Attiny85, Arduino, and ESP8266. For instance, when peripherals support is considered, STM32 outperforms the other types of hardware, as illustrated in the table. Since STM32 provides peripherals support capabilities, it is necessary to address power loss which may result from using peripherals in the sensor node. STM32 consists of larger memories compare to other microcontrollers. For example, STM32 consists of 64–128KB of flash memory, as compared to Attiny85, Arduino, and ESP8266, which consist of only 32KB flash memories [36,37,38]. STM32 also supports multi-tasking, which makes it subject to power loss if tasks management is not considered. Finally, STM32 is considered as one of the most common microcontrollers in several IoT and industrial applications [39,40]. Given the above description, this work considers STM32 as the ideal representative to apply the proposed power preservation approach in this work.

## 5. Optimizing Power Consumption of Sensor Nodes

This section describes the proposed approach for reducing power consumption on the microcontroller level and at the radio module level.

### 5.1. Reducing Energy Consumption at the Microcontroller Level

The STM32L152RE microcontroller is intended to perform multiple functionalities. Therefore, it runs on its high operation mode. By default, the STM32L152RE microcontroller operates at 32 MHz frequency (i.e., 33.3 DMIPS (Dhrystone Million Instructions per Second)). Several low-power modes can be implemented to save power when the microcontroller is idle. For instance, when the microcontroller is waiting for an external event, a lower mode can be used to save energy. In this work, we propose the sleep mode in which the microcontroller is totally turned off to preserve energy. To reactivate the microcontroller, the peripherals are kept active since they do not consume energy as much as the microcontroller. We use interrupts to reactivate the microcontroller upon the occurrence of an event.

To further optimize energy management and reduce the consumed power of the microcontroller to the minimal, we propose the enable/disable approach on a set of peripherals. We also control the system clock frequency in order to reduce energy utilization. In fact, the following proposed actions significantly contribute to reducing power consumption without affecting the intended functionalities of a sensor node. In this work, these actions are proposed for both the microcontroller unit as well as the peripherals:

- Decrease the clock frequency from 32 MHz to 65kHz after resetting the Reset and Clock Controller (RCC) configuration to the default reset state. RCC is used to manage multiple types of resets such as system reset and power reset. It provides flexible clock source choices which allows the designer to achieve both accuracy requirements and minimize power consumption. In this context, decreasing the clock frequency is the main reason for the considerable reduction in power consumption at the microcontroller level.
- Disable both the FLASH 64-bit access and FLASH prefetch buffer.
- Enable the FLASH power down and the fast WakeUp.

It is worth mentioning that reducing microcontroller clock frequency should be carefully analyzed for several reasons. First, active software processes should not be affected by reducing the clock frequency on a microcontroller. This means that it is essential to categorize the microcontroller tasks on the to decide into which extent clock frequency can be reduced. This may require task scheduling, which may also increase the complexity of the approach. Second, some peripherals require specific level of clock frequency to properly function. Hence, it is necessary to consider an overall view in which not only job scheduling is considered but also required peripherals. Finally, some microcontrollers are designed with fixed clock frequency. Therefore, reprogramming the microcontroller based in the reduced clock frequency requires tailoring the hardware, which can be a challenging task.

### 5.2. Reducing Energy Consumption at Radio Module Level

Energy consumption by the communication interface is one of the main factors of energy depletion in a wireless sensor node [41]. The purpose of the RDC mechanism is to periodically switch the radio of a sensor node *ON* and *OFF* in order to preserve energy. Hence, it extends the overall network longevity [4]. RDC has become an important mechanism in the design of WSNs. This is because the continuous use of the radio in a wireless sensor results in a great power consumption [8].

The RDC mechanisms are frequently referred to as MAC mechanisms/protocols. This is because they are usually implemented at the MAC layer. MAC protocols are typically categorized into two types, that are *scheduled-based protocols* and *contention-based protocols* [42]. The scheduled-based protocols allow nodes within a network to access network available resources (e.g., frequency, time) based on a pre-scheduled manner. With contention-based protocols, nodes compete to access a shared wireless medium. Each category has its own advantages and disadvantages. For example, an advantage of the scheduled-based protocols is that nodes consume less energy due to idle listening. This is because nodes sleep when they are not using their scheduled time slot. However, scalability is a significant issue in this type of protocol since scaling the network induces extra overheads due to resources rescheduling. On the other hand, simplicity and scalability are the main advantages of scheduled-based protocols. Inefficient use of power is the main disadvantage of this type of protocol. Based on the above discussion, the work in this paper aims at improving energy efficiency on contention-based protocols.

Some MAC protocols require fine time synchronization between nodes within a network. Synchronization is required to allow nodes to be aware of whether other nodes within the network are active or sleeping. Therefore, nodes are able to decide when to sleep to reduce energy consumption or when to be awakened to listen for incoming packets. The useful active window is a time period where all the nodes active frames overlap. It is recommended that the useful overlapping time be larger than a threshold that increases with the network size in order to ensure efficient management of all the application and control messages.

**Figure 3:**
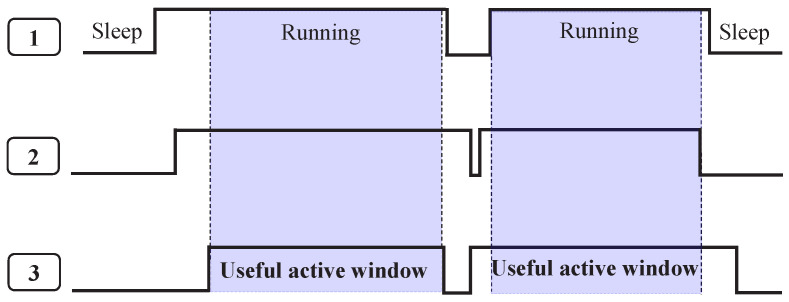
Synchronization activity and useful active window.

Figure 3 shows the useful active window. It shows three different nodes in their active and sleep state. The highlighted area within the figure represents the useful active window. In this area, nodes (1, 2, and 3) are overlapping in their running state. In fact, a good synchronization between nodes leads to keep the useful active window as close as possible to the total active period of the node. To illustrate, the basic idea of the RDC mechanism is to reduce idle listening since the radio can uselessly wait for a frame [9]. Hence, keeping the total active period of the node close to the useful active window within a network greatly improves the efficiency of the entire network.

For most applications, it is difficult to turn on the radio only when it is necessary. Hence, it is challenging to activate the radio only upon exchanging data packets (i.e., transmit or receive). This is because turning the radio to the active status at the right time for data reception is not obvious since nodes are not aware of incoming traffic in advance. It is worth noting that idle listening does not represent the only source of energy loss. There are other sources of power waste, such as collisions and control packet overhead [10]. Collisions in WSNs occurs when two nodes transmit their data through a communication medium simultaneously [9]. On the other hand, control packets represent packets that are sent or received for control purposes. This means that those packets do not contain any actual data to be conveyed [43]. Given that, minimizing idle listening using an RDC mechanism can introduce more control traffic side effects. It can also increase the collision rates, which leads to increased energy consumption. In fact, a good synchronization needs a frequent re-synchronization to manage the clock skews and, hence, an extra control traffic is required.

The shortening of time windows in the transmission and the reception using a RDC mechanism may increase the probability of collisions [10]. Given the fact that sensor nodes are generally battery powered, RDC mechanisms is considered as a key performance fact [44]. Various approaches are proposed in the literature with goals more ambitious. Note that the existing approaches differ mainly in their reliance on the synchronization of nodes within a network. This, in turn, leads into different complexity of implementation and requirements of hardware. In addition, RDC can also differ in other aspects, such as network density, increased end-to-end delay, and topology dependency.

In this work, we propose the ContikiMAC to improve power efficiency in a wireless sensor node. ContikiMAC is an RDC protocol available in Contiki OS. It represents a low-power listening-based protocol. It exhibits a very high-power efficiency [44]. We also employ the CCA technique. CCA employs RSSI of the transceiver radio to give an indication of the radio activity on the channel. Given that, we set a threshold to detect the activity in the channel. If RSSI is lower than a given threshold, then CCA indicates that the channel is clear of activity. Otherwise, CCA indicates that the channel is in use.

**Figure 4:**
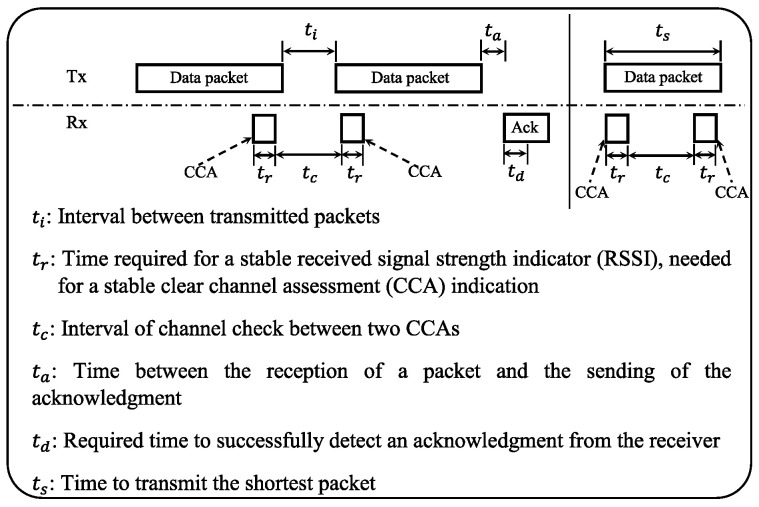
Timing constraints and definitions in ContikiMAC RDC mechanism.

Figure 4 depicts the timing constraints in which ContikiMAC operates. It shows how the necessary requirements for timing are defined. In the figure, the time interval between two transmitted packets is denoted by $t_{i}$. This time must be smaller than the channel check interval, which is denoted by denoted by $t_{c}$ (i.e., $t_{i}<t_{c}$). Time requirements are defined this way to guarantee that either the first or the second CCA is able to detect the packet transmission. Otherwise (i.e., $t_{c}<t_{i}$), a reliable detection of a transmitted packet is not possible by two CCAs.

The figure also shows the transmission time of the shortest packet. It is denoted in the figure by $t_{s}$, and must be larger than $2\times t_{r}+t_{c}$ (i.e., $t_{s}>2\times tr+tc$). When CCA detects the arrival of a packet, Contiki MAC keeps the radio ON to allow the reception of the full packet. Once the packet is successfully received, the receiver acknowledges it in response. Here, $t_{a}$ denotes the time between the reception of a packet and the sending of the acknowledgment. While $t_{d}$ denotes the required time to successfully detect an acknowledgment from the receiver. Thus, the timing constraints of ContikiMAC can be written as:

(1) $$t_{a}+t_{d}<t_{i}<t_{c}<t_{c}+2\times t_{r}<t_{s}.$$

Under IEEE 802.15.4 link layer, $t_{a}$, $t_{d}$, and $t_{r}$ in the inequality (1) are set as constants. First, $t_{a}$ is being set to 12 symbols where one symbol represents 4 ms. Therefore, $t_{a}$ is equal to 0.192 ms. Second, the reception of the acknowledgment can be reliably detected after the transmission of 4-byte long preamble and the 1-byte start of frame delimiter, which lasts $t_{d}$ = 40 = 0.16 ms. Finally, $t_{r}$ is defined to be 0.192 ms. Accordingly, the inequality (1) becomes:

(2) $$0.352<t_{i}<t_{c}<t_{c}+0.384<t_{s}.$$

The other variables (i.e., $t_{i}$, $<t_{c}$, and $t_{s}$) can be set as follows: Equation (2) gives a lower bound on $t_{s}>0.736$ ms. This defines a limit on the smallest packet size. For a bit rate of 250 kilobits per second (kbps) which is common in sensor communication, the size of packets must be at least 23 bytes. This is including preamble, start of frame delimiter, and length field. Consequently, the size of the packet data is 16 bytes. If ContikiMAC is required to send packet of size larger than the smallest one, the packet can be padded with null bytes to guarantee this requirement. Note that in the case of an IPv6 network layer packets with full IPv6 headers are always longer than the smallest ContikiMAC packet size on a IEEE 802.15.4 link layer. Using 6LoWPAN IPv6 header compression, packets can become smaller. However, a packet of size smaller than a given threshold, does not require the compression of the IPv6 header. Under ContikiMAC, $t_{i}$, $t_{c}$, and $t_{r}$ are set as follows: $t_{i}=0.4$ ms, $t_{c}=0.5$ ms, and $t_{s}=0.884$ ms. To evaluate the performance of ContikiMAC RDC compared to the NullRDC, we consider the application of the user datagram protocol (UDP) senders-receiver using STM32L152RE and the radio S2LP board.

**Figure 5:**
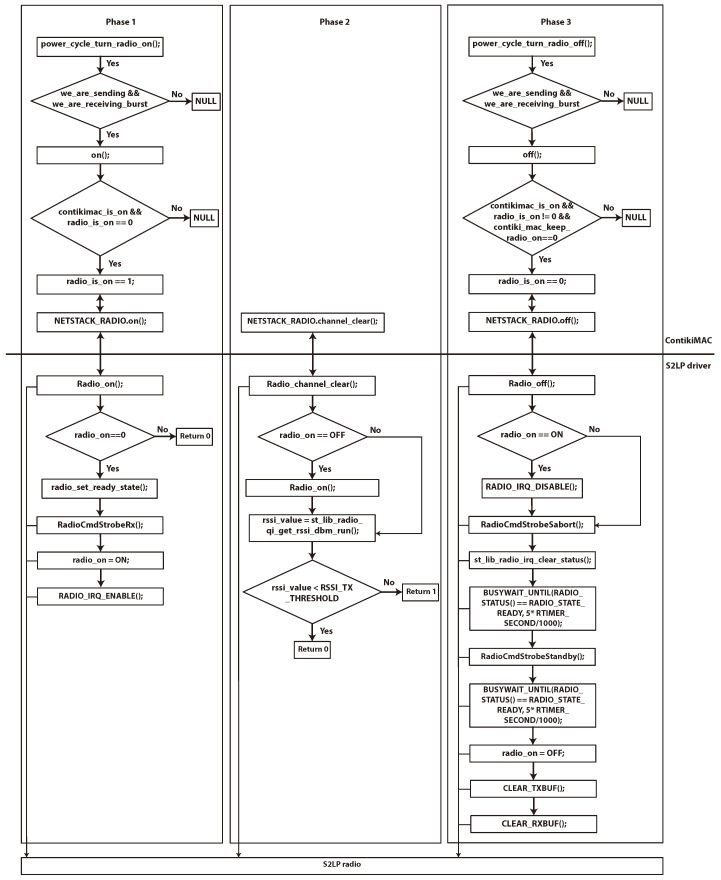
Relationship between contikiMAC RDC, S2LP radio deriver, and S2LP hardware.

Figure 5 presents the proposed process in this work. It shows the flowchart and the relationship between the ContikiMAC RDC mechanism, the S2LP radio driver, and the S2LP hardware. Typically, each RSSI check is constructed out of three different phases. These phases are required to prepare the radio module in a sensor node and to ensure proper communication process. As shown in the figure, the first phase consists of putting the S2LP radio in RX mode to be able to receive packets. To this end, the radio should pass by the ready state using the *radio_set_ready_state command()*. Then, we put the S2LP radio to receive mode RX using the *RadioCmdStrobeRx()* command. This phase is finished by setting the *radio_on* variable to *ON* and enabling the interrupt request (IRQ) through the *RADIO_IRQ_ENABLE()* command. Particularly, the radio driver access by RDC is checked. Additionally, the registration of the radio hardware is made in this phase.

In the second phase, we compute the RSSI value after ensuring that the S2LP radio is RX mode. If it is smaller than a predefined threshold, so the channel is clear. Otherwise, the channel is busy. The last phase is dedicated to the preparation of the radio to the next activity. For that reason, the radio IRQ is disabled, the radio is switched to *OFF* by moving its state to standby mode, the *radio_on* is set to *OFF* and both buffers for RX and TX modes are cleared. In fact, the flow chart in Figure 5 shows the details of each WakeUp process in each RSSI check in our proposed work.

## 6. Use Case

This section describes the experiments setup. In particular, it provides information about sensors components and physical setup. It also provides information about the utilized software and communication technologies.

### 6.1. Sensors’ Components and Setup

**Figure 6:**
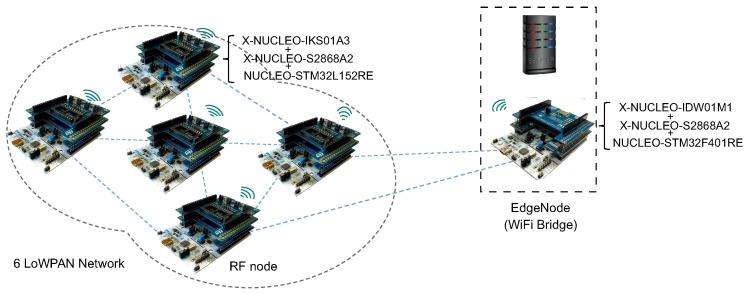
Hardware components of considered 6LoWPAN setup.

Figure 6 shows the simulated 6LoWPAN in this work. This network can be found in multiple IoT applications, such as smart grids and smart cities. The figure shows the utilized hardware and the layout of the network. It also shows two types of nodes—*sensor nodes* and *edge nodes* (i.e., Wi-Fi bridge). Each sensor node within the network is constructed out of three layers. The lower layer (e.g., hardware type in this case study [*NUCLEO-STM32L152RE*]) represents the microcontroller unit. The middle layer (e.g., *X-NUCLEO-S2868A2*) represents an expansion board based on S2LP RF transceiver used for sending data. The top layer (e.g., *X-NUCLEO-IKS01A3*) represents a temperature and humidity sensor, a microelectromechanical (MEMS) pressure sensor, a 3D MEMS magnetometer, an accelerometer, and a 3D MEMS gyroscope for measuring environmental aspects. Similarly, each Wi-Fi bridge is constructed out of three layers—a microcontroller (e.g., *NUCLEO-STM32F401RE*), a radio module (e.g., *X-NUCLEO-S2868A2*), and Wi-Fi board (e.g., *X-NUCLEO-IDW01M1*).

The communication within the simulated environment is performed using SubGHz technology through the use of IEEE 802.15.4 standard. SubGHz is used to improve the lifetime of the network since it consumes less energy compared to other wireless technologies. However, this approach can be extended to support different communication protocols based on the application and domain requirements. Additionally, the network is connected to the outside world via a single-board router. The modulation used in this use case is the frequency-shift keying (FSK). It has a base frequency centered at 868 MHz, a channel space, a frequency difference of 20 kHz, and a bit rate of 38.4 kbps. In this simulation environment, we use the IPv6 protocol as the communication protocol for the network layer. For routing communication packets, we use the routing protocol for low-power and lossy networks (RPL).

The experiments were conducted with six sensor nodes. Five nodes act as RF nodes to sense and communicate data, while the sixth node acts as a Wi-Fi bridge to connect the network to a remote server. In 6LoWPAN, nodes are randomly arranged within the network in different location with various distances ranging from 50 to 450 m. The closest sensor node to the Wi-Fi bridge is 420 m. On the other hand, the furthest sensor node is located about 670 m from Wi-Fi bridge. Data are delivered from the source node to the Wi-Fi bridge using different routs based on each source location.

### 6.2. Utilized Software and Communication Technologies

To perform the experiments in this simulation, we use UDP as a communication protocol for the transport layer. The network is connected to a Leshan lightweight machine-to-machine (LWM2M) server [45] with a Wi-Fi bridge. Leshan is a protocol from the Open Mobile Alliance (OMA) for M2M communication. It is built based on the Java programming language. It follows the client/server communication paradigm. Leshan has been chosen in this work since it is a lightweight model that focuses on IoT device management.

Leshan allows creating a local LWM2M server on a typical off-the-shelf computer. Using Leshan server (via its web page), it is possible to find the registered nodes and view a list of allocated resources/functionalities (e.g., temperature, humidity). The Wi-Fi bridge manages the transfer of packets in a completely transparent manner. Each node in the network is able to communicate directly with the LWM2M server. Leshan provides up to eight bidirectional communications channels from node to server or from server to node. In fact, eight represent the number of UDP sockets that the Wi-Fi module can open.

To register any node within the network, each node sends registration information to the LWM2M server. This allows users to display the information collected by different nodes on the IoT network. The microcontroller units on a sensor node and a Wi-Fi bridge are programmed to manage other modules and perform data acquisition. They are also used to analyze and ensure connectivity with the network and with the SubGHz radio module. Each sensor node has a sensor module, and each Wi-Fi bridge has a Wi-Fi module. Sensor nodes and Wi-Fi bridges in this work are implemented with two Nucleo STM32 boards integrated with expansion boards.

**Figure 7:**
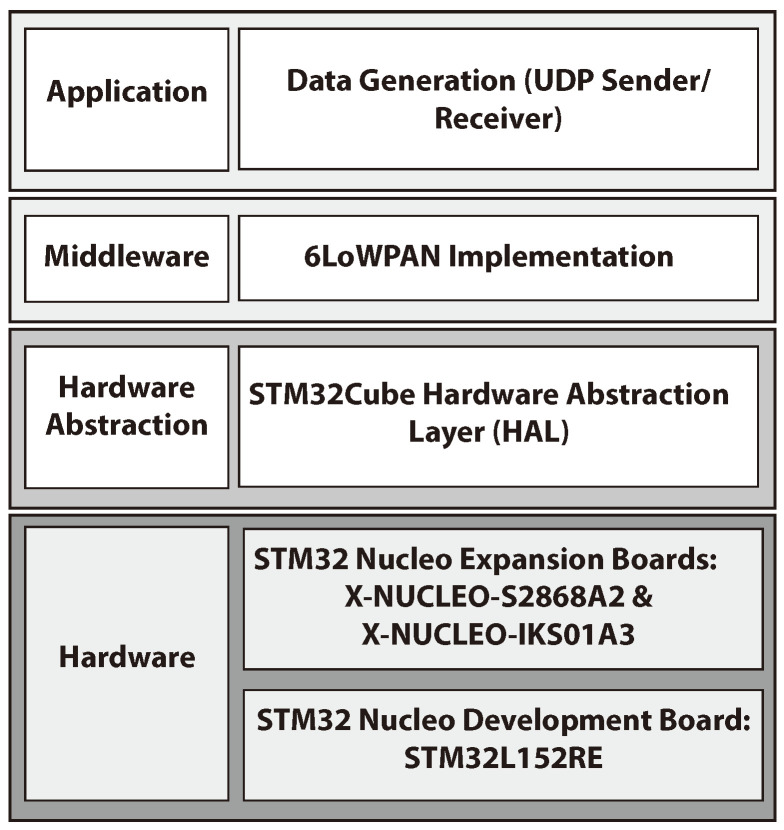
X-CUBE-SUBG2 architecture.

To develop software on STM32 Nucleo boards, there is a set of libraries called STM32Cube. Those libraries are available in various versions depending on the microcontroller type. STM32Cube [46] is a development framework designed to provide a set of application programming interface (API). It supports the development of standard APIs for all the STM32 microcontrollers, which enhances the portability of the application layer. Figure 7 illustrates the architecture X-CUBE-SUBG2, which is considered as an expansion set of programs for STM32Cube. X-CUBE-SUBG2 consists multiple software layers that are used by the application to access and use the S2LP expansion board. These layers are defined as follows:

- Hardware layer: this layer consists of board support package, which consists of set of libraries to facilitate the peripherals on the STM32 Nucleo board. It provides set of APIs that are used to interface with specific peripherals such as user buttons and LED. The different expansion boards (i.e., X-NUCLEO-S2868A1, X-NUCLEO-S2868A2, X-NUCLEO-S29 15A1) consist of set of programming libraries to manage the hardware components.
- Hardware abstraction layer: this layer is used to support the upper layers. It provides multiple instances of generic set of APIS to support the interaction with the upper layers. HAL includes some tailored and generic extension of APIS that are built based on a common model. Hence, other layers such as the middleware can normally function without the need of extra hardware configurations. In fact, this contributes to improving code reusability and facilitate portability.
- Middleware: this layer consists of set of APIs to ease the implementation of 6LoWPAN. It provides generic software libraries to facilitate the integration of application with the lower layers.
- Application layer: this layer provides 6LoWPAN communication for mesh network. In this work it is tailored to serve with Contiki-based applications. The layer also provides features for Client/Server UDP communication.

## 7. Evaluation

This section provides insights on evaluating the effectiveness of the proposed approach in this work. It shows the obtained results of the use of the sleep mode comparing with the normal mode using STM32L152RE as the base microcontroller. It also shows the advantages of using ContikiMAC as RDC mechanism instead of NullRDC.

### 7.1. Network Settings and Parameters

**Figure 8:**
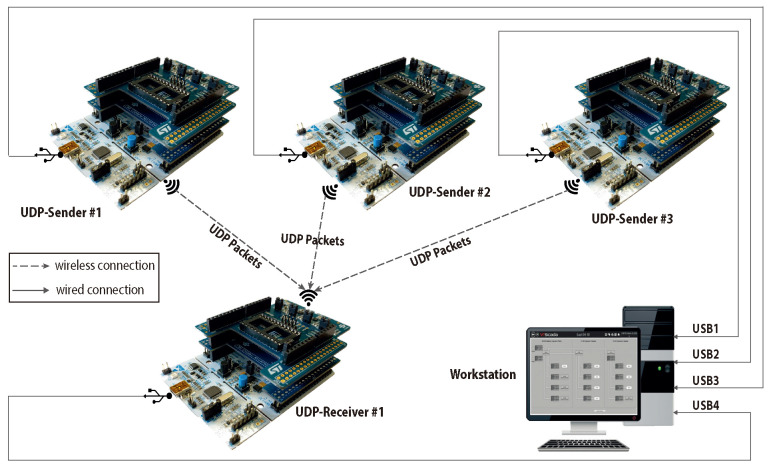
Considered application using ContikiMAC RDC.

To evaluate the proposed approach, we compare the performance of the ContikiMAC RDC mechanism against the performance of NullRDC (which is the default mechanism in Contiki OS). We implement multiple senders/receivers nodes to read and exchange sensing data. The sensor nodes are equipped with *NUCLEO-STM32 L152RE*, *X-NUCLEO-S2868A2*, and *X-NUCLEO-IKS01A3*. Figure 8 illustrates the network layout and the utilized devices in the performed experiments. The figure represents a subset of the illustrated network in Section 6. This subset represents a specific sensing application that is designed to observe and communicate environmental data (i.e., temperature, humidity). The figure shows three sender nodes and one receiver. All the nodes are connected to a workstation to exchange control data. Additionally, sender nodes are connected to the receiver node, which is designated as an aggregation node. Throughout the experiment, nodes exchange sensing data wirelessly using UDP packets.

To analyze the power performance based on the proposed approach, we utilize *STM32CubeMonitor-Power*. *STM32Cube Monitor-Power* is an analysis tool that allows developers to acquire power measurements and display them using graphical interfaces. Table 2 shows the configured units in the experiments. It first shows the parameters (i.e., *SEND_INTERVAL* and *SEND_TIME*) that are configured for sending UDP packets. It also shows the parameters setting for *STM32CubeMonitor-Power*. To illustrate *STM32Cube Monitor-Power* is set to continuous mode (i.e., *Acquisition Time* = *∞*). This mode sets the monitor to continuously read all power measurements. The *Sampling Frequency* on the monitor is set to 50 kHz. The Carrier Sense Multiple Access (CSMA) protocol is set to be disabled in this experiment. CSMA is disabled since sensor nodes are communicating using UDP packets.

### 7.2. Sleep Mode Evaluation

**Figure 9:**
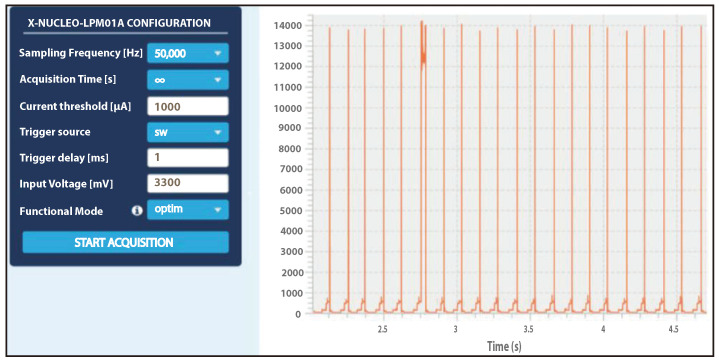
Electrical power consumption of STM32L152RE in sleep mode under 65 KHz.

The following describes the current consumption evaluation of the sleep mode using STM32L152RE microcontroller unit. First, we evaluate the energy consumption of the NUCLEO- STM32L152 RE used by the sensor nodes. We use the X-NUCLEO-LPM01A boards to retrieve the current consumption measurement of the microcontroller unit. Figure 9 shows the power measurements using *STM32CubeMonitor-Power*. The figure shows that when the microcontroller unit enters the sleep mode using 65 KHz as a system clock frequency instead of 32 MHz, the electrical power consumption decreases from around 14 μA to a few μA. This is considered to be significant power reduction especially with such a constrained device. Therefore, it can be concluded that the proposed sleep mode outperforms the normal mode in terms of energy consumption. Consequently, the proposed sleep mode enhances not only the lifetime of the battery-powered device but also the lifetime of the entire network.

### 7.3. RDC Evaluation

In the following, we evaluate the use of ContikiMAC as RDC mechanism instead of NullRDC. We consider two important factors in the evaluation process. The first is the amount of the consumed power for each sensor node when the node acts as a sender or as a receiver. The second factor, which is as important as the amount of consumed power is the reliability of communication. We measure reliability by the percentage of *packet drop rate*. Additionally, we consider testing the approach with continuous running measurements and with separate time period measurements.

#### 7.3.1. Evaluating Power Consumption

**Figure 10:**
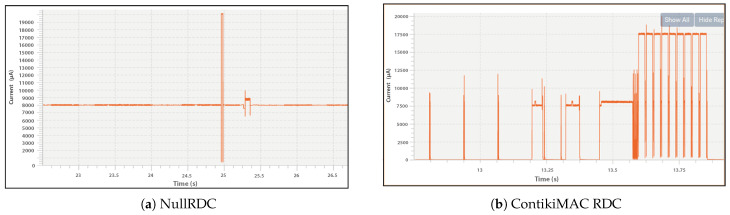
Power consumption of S2LP radio.

This subsection describes the evaluation of the consumed power in the case of continuous running and separate time period measurements. For measuring the continuous running, we run the sensor node for several seconds. Based on the running time, we observe the consumed energy by S2LP radio of the sensor node. Figure 10a shows the amount of consumed power when NullRDC is used. It shows that with NullRDC the S2LP radio is always in RX mode with a continuous power consumption of around 8 μA. This results in a significant power loss of the sensor node, which results in other issues, such as coverage holes and data loss.

On the other hand, Figure 10b shows the amount of consumed power when ContikiMAC is used. It can be seen from the figure that a significant amount of energy has been preserved as compared to NullRDC approach. This is because when using ContikiMAC, the S2LP radio is in RX mode only to perform CCA. In fact, when we compare the NullRDC approach with the proposed ContikiMAC approach, we observe that using ContikiMAC contributes to saving up to 50% of the wasted energy.

To further evaluate the proposed ContikiMAC approach, we consider separate time period measurements. For this test, we designed three different communication scenarios. These scenarios depend on the number of communicating nodes and they are: (a) one sender one receiver, (b) two senders one receiver, and (c) three senders one receiver. For each scenario, we measure the consumed power with NullRDC approach as compared to the ContikiMAC approach. Additionally, for each scenario, we assess the current consumption average of the radios of both the sender and the receiver for 4 periods (1, 5, 10, and 20 min). The measurements are performed using the energy monitor board (i.e., X-Nucleo-LPM01A) in conjunction with the STM32CubeMonitor power.

Different distances are considered for each one of the above described scenarios. For the first scenario (a), the distance between the sender node and receiver node is 100 meters. For the second scenario (b), the average distance between the sender node and receiver node is 220 meters. For the third scenario (c), the average distance between the sender node and receiver node is 370 meters. To rigorously evaluate the results for each scenario, the experiments were repeated 10 times for each time period. It is worth mentioning that since the experiments were performed in an open environment the exchanged data were subject to external interference factors.

**Figure 11:**
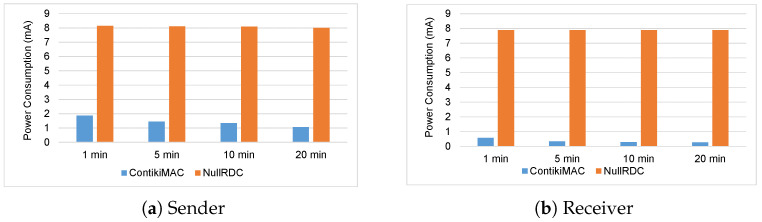
Power consumption of the sensor node based on first scenario.

Figure 11 shows the current power consumption of a sensor node based on the first scenario. The graph (a) shows the power consumption of the sensor node when it acts a sender. It shows that for all for four periods the average power consumption with the ContikiMAC approach is about 1.4 μA. On the other hand, the average power consumption when the NullRDC approach is used is about 8.1 μA. The graph (b) shows the power consumption of the sensor node when it acts a receiver. From the figure, it can be seen that with all the 4 periods, when the ContikiMAC approach is used the average power consumption is around 0.4 μA. As opposed to ContikiMAC approach, the graph (b) shows significant power consumption with average of 7.9 μA when the NullRDC approach is used.

**Figure 12:**
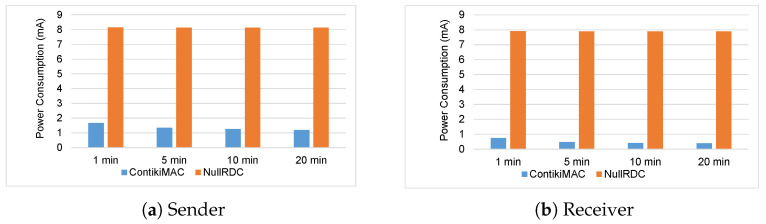
Power consumption of the sensor node based on second scenario.

**Figure 13:**
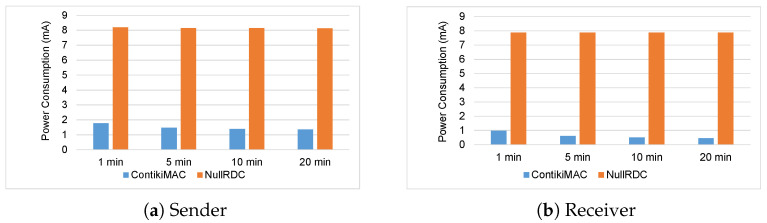
Power consumption of the sensor node based on third scenario.

Similarly, Figure 12 and Figure 13 show the power consumption of a sensor node based on the second and third scenarios, respectively. Generally, both figures show that the ContikiMAC approach significantly reduces power consumption as compared to NullRDC approach. The graphs (a) in both figures show a similar average in power consumption of 1.5 μA when the sensor acts as a sender. Additionally, graphs (b) in both figures show slight increase in the average of the consumed power (around 0.6 μA) with ContikiMAC approach. This is normal because the second scenario the receiver obtains data from two different senders. While, in the third scenario, the receiver obtains data from three different senders.

#### 7.3.2. Evaluating Communication Reliability

Based on the discussion provided in Section 2, it is obvious that WSNs require practical solutions to extend the lifetime of a sensor node through power management techniques. However, any proposed solution must not compromise the basic functionality of sensor nodes which is sensing and transmitting data. Therefore, to complete the evaluation of the proposed approach in this work, we measure the reliability of communication. For this experiment, we use the above described scenarios. For each scenario, we assess packet drop rate for four different time periods (1, 5, 10 and 20 min).

Table 3, Table 4 and Table 5 show the results of the packet drop rate based on each scenario. For instance, Table 3 shows that with the proposed ContikiMAC there is a trivial packet drop rate for the third and fourth measurement periods. Table 4 also shows a similar drop rate with little increase in the packet drop rate at the last measurement. On the other hand, Table 5 shows that the proposed ContikiMAC approach outperforms the NullRDC approach at all measurement periods. The increased packet drop rate in scenario 3 is due to two reasons. First, is that without CSMA, two or more transmissions can be started at the same time, which may cause collision. Secondly, is that the current radio driver of NullRDC does not support the transmission of acknowledgement as a confirmation of packet reception. It is worth noting that, for both NullRDC and ContikiMAC, in scenarios 2 and 3 (with two and three senders, respectively), it may happen that there are few packets, which are lost. Normally, this is due to the fact that the two/three senders start to transmit packets at the same time. We can solve this problem by enabling CSMA.

By evaluating the gain provided by ContikiMAC compared to NullRDC in percentage, one can see that ContikiMAC remarkably reduces the consumption average of both the sender and receiver for all the three scenarios. Consequently, the lifetime of the entire network is optimized especially when the network heavily depends on battery-powered nodes. In addition, the gain increases as a function of the time since the RPL (Routing Protocol for Low-Power and Lossy Networks) packets will be rarely sent with the time. Additionally, the proposed approach also performs well in terms of reliability and packets delivery. In fact, the running experiments in this work have shown that the practical proposed steps in this work greatly improves the sustainability of WSNs.

#### 7.3.3. Overall Evaluation

In order to provide an overall evaluation view of the proposed approach, the work in this paper is compared with related work that is described in Section 3. Table 6 provides the evaluation criteria that are specific to the aspects that this work is addressing. The evaluation criteria are listed on the top row. The *Protocols Support* specifies whether if the approach is able to be adopted/applied by different communication protocols or not. The *Transmission (Flexible Length)* indicates if the approach imposes specific restrictions on the length of transmitted data packets. *Low Complexity* shows if the proposed work induces complexity, such as overhead or synchronization requirements. Finally, the *Multi-Domain Support* indicates if the work is flexible to be adopted in multiple applications and domains. From the table, it can be seen that our proposed approach can be extended to support multiple communication protocols. It can also be applied in different IoT applications since it does not impose any domain-specific restrictions. Additionally, our work does not restrict the length of the transmitted packets, as long as the communication protocol supports the length. Finally, the work does not introduce synchronization requirements, which lowers the complexity of the approach and enhances its flexibility.

## 8. Conclusions

In this work, we have presented an approach for reducing power consumption on a Contiki-based sensor node. The main objective of the work is to improve the lifetime of a WSN. In this study, we first categorize the main source of energy loss in a sensor node. We then describe energy conservation mechanisms at two levels. The first mechanism targets maintaining energy at the microcontroller unit in a sensor node. Therefore, we propose multiple tailoring points to reduce the amount of utilized energy. In the second mechanism, we propose an approach to deal with power loss at the radio module level. We tailor the default RCD mechanism in Contiki OS and we replace it with another mechanism known as ContikiMAC. We tested the approach using real-life experiments with multiple interconnected sensor nodes. Based on that, we measure power consumption and communication reliability. The results show that the proposed approach has the ability to save significant amount of energy without affecting the reliability of communication. In the future, we plan to extend the approach to include other operating systems and different WSNs and system architectures. We also plan to generalize the approach to work in heterogeneous WSNs environments, such as LoRaWAN and SIGFOX.

## Figures and Tables

**Table 1:** Comparison between common used microcontrollers [36,37,38,39,40].

	Peripherals Support	Memory Capacity	Multitasking	Commonality in IoT and Industrial Apps.
**STM32**	Capable	High	High	Common
**Attiny**	Constrained	Constrained	Constrained	Moderate
**Arduino**	Limited	Limited	Limited	Common
**ESP8266**	Constrained	Constrained	Limited	Moderate

**Table 2:** Configured parameters.

Unit	Parameter	Configuration
UDP Packets	SEND_INTERVAL	25*CLOCK_SEND
	SEND_TIME	5000 % SEND_INTERVAL
STM32CubeMonitor-Power	Acquisition Time	*∞*
	Sampling Frequency	50 kHz
CSMA	-	Disabled

**Table 3:** One sender one receiver—packet drop rate %.

	1 min	5 min	10 min	20 min
ContikiMAC	0	0	0.85	0.85
NullRDC	0	0	0	0

**Table 4:** Two senders one receiver—packet drop rate %.

	1 min	5 min	10 min	20 min
ContikiMAC	0	0	0.84	1.28
NullRDC	0	0	0	0

**Table 5:** Three senders one receiver—packet drop rate %.

	1 min	5 min	10 min	20 min
ContikiMAC	0	0	0.57	0.71
NullRDC	3.33	1.72	1.70	2.28

**Table 6:** Proposed approach compared to work in literature.

	Protocols Support	Transmission (Flexible Length)	Low Complexity	Multi-Domain Support
**WakeUp Receiver [25]**	√	x	√	x
**Channel Partitioning [24]**	√	x	x	√
**Multi-Hop Trans. [29]**	x	√	√	x
**WSSs with BLE [30]**	x	x	√	√
**WSSs with ZigBee [31]**	x	x	√	√
**Proposed Work**	√	√	√	√

## Data Availability

Not applicable.
